# Integration of Single-Cell and Bulk Transcriptomes to Identify a Poor Prognostic Tumor Subgroup to Predict the Prognosis of Patients with Early-stage Lung Adenocarcinoma

**DOI:** 10.7150/jca.105926

**Published:** 2025-01-21

**Authors:** Zijian Shi, Linchuang Jia, Baichuan Wang, Shuo Wang, Long He, Yingxi Li, Guixin Wang, Wenbin Song, Xianneng He, Zhaoyi Liu, Cangchang Shi, Yao Tian, Keyun Zhu

**Affiliations:** 1 Department of Thoracic Surgery, Ningbo Medical Center Lihuili Hospital, Ningbo University, Ningbo, Zhejiang Province, 315040, China.; 2 Department of Lung Cancer Surgery, Tianjin Medical University General Hospital, Tianjin, 300052, China.; 3 Department of Physiology and Pathophysiology, School of Basic Medical Sciences, Tianjin Medical University Cancer, Tianjin, 300070, China.; 4 Anhui Chest Hospital, Anhui Medical University Clinical College of Chest, Hefei, Anhui Province, 230022, China.; 5 The First Department of Breast Cancer, Tianjin Medical University Cancer Institute and Hospital, National Clinical Research Center for Cancer, Tianjin 300060, China; 6 Department of General Surgery, Tianjin Medical University General Hospital, Tianjin Key Laboratory of Precise Vascular Reconstruction and Organ Function Repair, Tianjin General Surgery Institute, Tianjin, 300052, China.; 7 Immunology Department, Key Laboratory of Immune Microenvironment and Disease (Ministry of Education), Tianjin Medical University, Tianjin, 300070, China.; 8 Health Science Center, Ningbo University, Ningbo, Zhejiang Province, 315040, China.

**Keywords:** Lung Adenocarcinoma, Single-cell RNA sequencing, Tumor microenvironment, Biomarkers, Prognostic model

## Abstract

**Background:** Single-cell RNA sequencing (scRNA-seq) has emerged as a pivotal technology for investigating novel therapeutic targets in cancer. Despite its significance, there remains a scarcity of studies utilizing this technology to address treatment strategies specifically tailored for early-stage lung adenocarcinoma (LUAD). Consequently, this study aimed to investigate the tumor microenvironment (TME) characteristics and develop a prognostic model for early-stage LUAD.

**Methods:** The markers identifying cell types were obtained from the CellMarker database and published research. The SCEVAN package was employed for identifying malignant lung epithelial cells. Single-cell downstream analyses were conducted using the SCP package, encompassing gene set enrichment analysis, enrichment analysis, pseudotime trajectory analysis, and differential expression analysis. Calibration curves, receiver operating characteristic curves, and decision curve analysis were employed to assess the performance of the prognostic model for LUAD. Reverse transcription-quantitative polymerase chain reaction (RT-qPCR), western blot, cell transfection, cell proliferation, and cell invasion assays were performed to validate the expression and biological function.

**Results:** Seven cell types were distinguished in the scRNA-seq dataset through the utilization of cell markers documented in published literature. Four subpopulations of early-stage LUAD tumor cells exhibited a high degree of heterogeneity. The prognostic model constructed by *PERP* and *KRT8* showed a great prediction for distinguishing the early-stage LUAD and normal tissues. The validation of *PERP* and *KRT8* expression levels was carried out through both RT-qPCR and western blot analyses. Eventually, *in vitro* experiments, including CCK8, colony formation, EdU, and transwell assays, confirmed that *KRT8* and *PERP* could promote LUAD cell proliferation and migration.

**Conclusions:** Our study provided a comprehensive characterization of the TME in LUAD through integrative single-cell and bulk transcriptomic analyses. We identified dynamic transitions from normal epithelial cells to tumor cells, revealing the heterogeneity and evolution of malignant LUAD cells. The novel prognostic model based on KRT8 and PERP demonstrated robust predictive performance, offering a promising tool for early-stage LUAD risk stratification. Functional experiments further confirmed that KRT8 and PERP promote tumor proliferation and migration, providing new insights into their roles as therapeutic targets.

## Introduction

Globally, lung cancer remains the leading cause of cancer-related mortality^1^, ^2^. Even in its early stages, the prognosis for patients with lung cancer is relatively unfavorable when compared to other prevalent cancers, like colon, breast, and prostate cancer^3^. Lung adenocarcinoma (LUAD) stands out as the most frequently diagnosed pathological type of lung cancer in clinical practice^4^, ^5^. Recently, many patients with LUAD have been detected by low-dose computed tomography at earlier pathological stages^4^, ^6^. Subsequently, a growing number of instances of early diagnoses in LUAD have underscored the necessity for novel personalized early treatment approaches. The effectiveness of such strategies is contingent upon an enhanced comprehension of the molecular and cellular processes occurring during the early stages of LUAD.

Tumor microenvironment (TME) is essential for tumor development, and many studies have shown that TME is a vital source of intra-tumor heterogeneity (ITH)^7^-^10^. Therefore, it is necessary to characterize TME and ITH at different stages of LUAD comprehensively. Moreover, most studies investigating LUAD transcriptome profiles have been based on bulk RNA techniques, which may not be able to detect the cellular diversity and molecular complexity of tumor cells. The advent of scRNA-seq has proven to be a potent tool for comprehensively unraveling the cellular composition of resected non-small cell lung cancer (NSCLC) specimens^11^, ^12^. This technological advancement has successfully overcome previous technical limitations, providing a deeper insight into the heterogeneity inherent in NSCLC^11^, ^13^. Consequently, a more profound understanding of the TME in LUAD could contribute to unveiling the mechanisms underpinning tumor progression and onset. Moreover, it holds promise in identifying novel biomarkers and potential therapeutic targets for LUAD.

We thoroughly analyzed the TME in LUAD and developed a prognostic model that includes *KRT8* and *PERP* in this study. Additionally, the evolution of lung epithelial cells into tumor cells exhibited dynamic alterations, highlighting a nuanced understanding of this transitional process. This study also revealed high heterogeneity among tumor cells, further emphasizing the diverse nature of these cancerous cells in LUAD. Finally, in order to validate the biological function of two genes, *in vivo* experiments were conducted. Our discoveries have yielded fresh perspectives on the development of biomarkers for early-stage LUAD.

## Materials and Methods

### Data collection and preprocessing

The single cell-RNA expression profiles were downloaded from the gene expression omnibus database. Three pairs of early-stage LUAD and normal samples in the GSE117570 dataset were included in the following analysis. We leveraged the TCGAbiolinks package to retrieve bulk RNA expression data and associated clinical information for 427 patients diagnosed with early-stage lung adenocarcinoma (LUAD) via R4.2.2. The 321 early-stage LUAD samples were extracted from the GSE72094 dataset.

The Cancer Genome Atlas (TCGA)-LUAD dataset served as the training dataset, with GSE72094 utilized as the validation dataset.

To obtain high-quality single cells, we performed the following criteria to remove low-quality single cells and potential doublet: 1. The genes detected per cell was >300. 2.UMIs >500. 3. The portion of the mitochondria-expressed gene was <40%. Furthermore, the doubletfinder package was conducted to remove potential doublet. Moreover, to remove the batch effect, a harmony package was conducted. The gene expression value of TCGA-LUAD samples was transformed into TPM to reduce the effect of sequencing depth and gene length.

To ensure consistency and reliability, we applied strict inclusion and exclusion criteria for patient selection. Inclusion criteria included patients diagnosed with early-stage LUAD (stage I or II), no history of neoadjuvant chemotherapy or radiotherapy, and no history of other tumors prior to diagnosis. Patients without complete prognostic information or expression profiles were excluded. The detailed clinicopathological parameters of patients were summarized and are provided in Additional File 1: Table S1.

### CNV analysis

SCEVAN is a simple and effective package to distinguish the tumor and normal cells for the scRNA-seq dataset. The expression matrix of lung alveolar epithelial cells was extracted and subjected to copy number variation (CNV) analysis to identify malignant lung alveolar epithelial cells using the SCEVAN package.

### Single-cell downstream analysis

The SCP package (https://github.com/zhanghao-njmu/SCP) was used to infer the development of the potential cell lineage by the slingshot algorithm. Furthermore, the biological dynamic changes, including gene expression and ontology, were analyzed for the cell lineage.

### Transcription factor analysis

To investigate the heterogeneity of four distinct tumor subgroups further, the pySCENIC analysis was performed by the Linux system. The expression matrix of four subgroups was extracted and transformed into loom format through Python 3.9. After the standard three steps (GRN, cisTarget, and AUCell) of pySCENIC, the output file was obtained and visualized using R.4.2.2.

### Construction of the prognostic model

The C3 subgroup markers were obtained by the RunDEtest() function in SCP. Eighty-nine genes with log2 fold change >1 and P-value <0.05 were considered markers of the C3 subgroup. A total of 4235 differential-expressed genes in LUAD tissue (compared to normal tissues) were calculated using the edgeR package. The intersection of the genes resulted in the identification of 25 genes. The least absolute shrinkage and selection operator (LASSO) algorithm was employed to filter critical genes for LUAD prognosis. Multivariate COX analysis was conducted to calculate the coefficients. The discriminative performance of the model for predicting 1-, 3-, and 5-year overall survival (OS) rates was assessed by using time-dependent receiver operating characteristic (ROC) curves. High- and low-risk patient groups were stratified using the best cutoff value determined by the survminer package in R, which selects the optimal threshold based on the maximized log-rank statistic for survival analysis. Kaplan-Meier curves were employed to assess survival outcomes in both high- and low-risk groups. To assess the model's ability to predict accurately, we employed calibration curves using the bootstrap method with 500 resamples.

### Potential drugs for patients with high-risk

The genes exhibiting differential expression between patients with high and low risk in the TCGA-LUAD dataset were estimated by edgeR package. Subsequently, the hallmark gene sets were used to annotate signaling changes in the patients with high risk. Furthermore, the potential drugs for patients with high risk were estimated by the connectivity map (CMAP) database. Specifically, we uploaded 300 differential genes between high and low groups. Moreover, the CMAP database calculated and returned drugs with a value between -1 to 1. Smaller values indicate more effective drugs.

### UALCAN

UALCAN (http://ualcan.path.uab.edu/analysis.html) serves as an online platform offering comprehensive analysis of gene expression, utilizing data from TCGA^14^. To further investigate *KRT8* and *PERP* expression, we compared their transcription levels in lung cancer and normal tissues using data from the UALCAN database.

### Human protein atlas

Human Protein Atlas (https://www.proteinatlas.org) includes immunohistochemistry-based expression data^15^. Immunohistochemical analysis of *KRT8* and *PERP* protein expression was conducted in normal lung and tumor tissues.

### Tissue specimens

Twenty cancerous and adjacent non-cancerous lung tissue pairs were collected at Ningbo Medical Center Lihuili Hospital. Histological examination confirmed all tumor samples as lung cancer. Written informed consent was acquired from all involved patients. This study was approved by the Ningbo Medical Center Lihuili Hospital Ethics Committee and was consistent with the ethical guidelines of the Helsinki Declaration.

Samples were selected based on the following criteria: Patients with histopathologically confirmed early-stage lung adenocarcinoma (LUAD) were included, provided they had not undergone neoadjuvant chemotherapy or radiotherapy. Patients with other malignancies, severe systemic diseases, or inadequate tissue quality—such as insufficient tumor content or degraded RNA—were excluded.

### RNA extraction, cDNA synthesis, and RT-qPCR

Total RNA was isolated from both frozen and surgically resected lung tissues using TRIzol reagent (Invitrogen, USA) according to the manufacturer's instructions. cDNA was synthesized by reverse transcription of RNA using PrimeScript RT Master Mix (TaKaRa, Japan). RT-qPCR was conducted using pre-designed primers with a Bio-Rad CFX96 system. The and siRNAs oligonucleotides are listed in Additional file 1: Table S2.

### Cell culture, transfection and cell function assays

Seven lung adenocarcinoma cell lines (H1650, A549, H1975, H3122, H2228, H1299 and PC-9), one normal lung cell line (BEAS-2B) were obtained from the Type Culture Collection of the Chinese Academy of Sciences. The cells were cultured in RPMI-1640 medium (Gibco, USA) containing 10% fetal bovine serum (FBS; PAN-Seratech) and 1% penicillin-streptomycin (PS; HyClone) in a 5% CO_2_ and humidified atmosphere at 37 °C. The siRNAs of *KRT8* and *PERP* constructs were synthesized by RiboBio (Guangzhou, China). The protocol of cell transfection was conducted as previously described^16^. Cell proliferation ability was assessed using CCK8 assays, colony formation and EdU assay. Cell migration and invasion were evaluated using transwell and wound healing assays, respectively. All experiments were performed as previously described^17^, ^18^.

### Western blot and antibodies

Cells were collected and washed with cold PBS and lysed on ice using radioimmunoprecipitation assay buffer (Solarbio) supplemented with 1 mM protease inhibitor at 4 °C for 30 min. The collected protein was denatured at 95 °C in a water bath for 10 min. The protein concentration was determined following the manufacturer's instructions using a bicinchoninic acid protein analysis kit (Solarbio). Equal amounts of proteins were separated using SDS-PAGE. Proteins were then transferred to polyvinylidene difluoride membranes, blocked with 5% bovine serum albumin, and incubated with primary and secondary antibodies. The details of the antibodies used in this study are presented in Additional File 1: Table S3.

### Immunohistochemistry (IHC)

IHC was performed using standard protocols. Tissue sections underwent antigen retrieval in citrate buffer (pH 6.0) using a pressure cooker for 15 minutes. Endogenous peroxidase activity was blocked with 3% hydrogen peroxide for 10 minutes, followed by blocking with 5% bovine serum albumin (BSA) for 30 minutes at room temperature. Primary antibodies against KRT8 and PERP were applied overnight at 4°C. After washing, HRP-conjugated secondary antibodies were incubated for 30 minutes at room temperature. Staining was visualized with DAB and counterstained with hematoxylin. Finally, sections were dehydrated and mounted for evaluation. To ensure diagnostic consistency, all samples were independently reviewed and confirmed by two experienced pathologists.

IHC results were evaluated independently by two experienced pathologists using a semi-quantitative scoring system: Staining intensity: Scored from 0 to 3 (0: no staining, 1: weak, 2: moderate, 3: strong). Percentage of positive cells: Scored as follows: 0 (0%), 1 (<25%), 2 (25-50%), 3 (>50%). The final IHC score was calculated by multiplying the intensity and percentage scores (range 0-9). Discrepancies between the two pathologists were resolved through discussion until consensus was reached.

### Statistical analyses

The RT-qPCR results were subjected to statistical analysis using Prism 8 software (GraphPad Software, CA) and are presented as mean ± standard deviation (SD) for at least three individual experiments. The statistical significance of differences was determined with the unpaired, two-tailed student *t*-test, and *P* < 0.05 was considered statistically significant. Other statistical analyses were performed using R studio software 4.2.2.

## Results

### Identification of cell types

**Figure 1:**
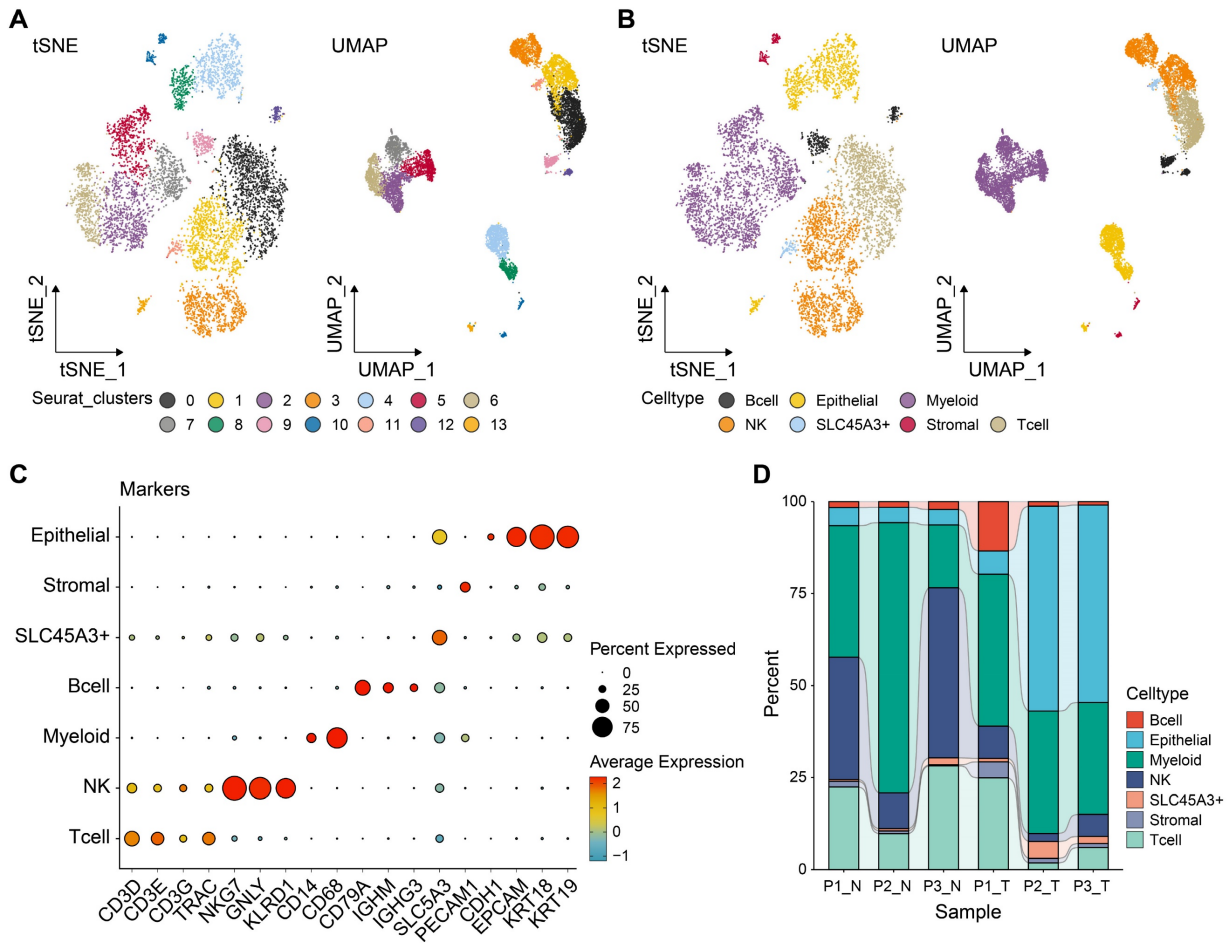
** Distinct cell types in LUAD were identified through single-cell sequencing.** The cell clusters (A) and cell types (B) in LUAD samples demonstrated using the uniform manifold approximation and projection (UMAP) and t-distributed stochastic neighbor embedding (TSNE) plots according to their featured gene expression profiles. (C) Dot plot displaying the expression level of marker genes for annotating the cell types. (D) The cell type portions of normal and tumor samples in scRNA-seq dataset.

Following stringent quality control steps implemented with the Seurat package, a total of 8,661 individual cells were identified for further analysis. Subsequently, following scRNA-seq analysis, our data revealed 14 unique cell populations through clustering with principal component analysis (PCA) at a resolution of one. Data visualization through two-dimensional reduction techniques, namely t-distributed stochastic neighbor embedding and uniform manifold approximation and projection (UMAP), demonstrated distinct separation of various cell clusters, as depicted in Fig. 1A. Utilizing markers obtained from the cell marker database, cell types were further clarified, leading to the categorization of all cells into eight groups: T, NK, B, epithelial, myeloid, stromal, and SLC45A3+ cells, depicted in Fig. 1B. SLC45A3+ cells were identified as a group of cells lacking significant features of known cell markers. These cells were named based on the prominent expression of the gene SLC45A3. The markers employed for annotating cell types are illustrated in Fig. 1C. For instance, *NKG7*, *GNLY*, and *KLRD1* are indicative of NK cell identity. As shown in Fig. 1D, the cellular proportions of various cell types differed significantly between normal and tumor samples. For example, the proportion of NK and T cells was found to be higher in normal samples compared to tumor samples., suggesting immune dysregulation in TME. Moreover, tumor samples showed a significantly higher proportion of epithelial cells compared to normal samples. This may be caused by high cell proliferation of tumor cells. Seven cell types were identified for further analysis.

### The cell trajectory of LUAD epithelial cells

**Figure 2:**
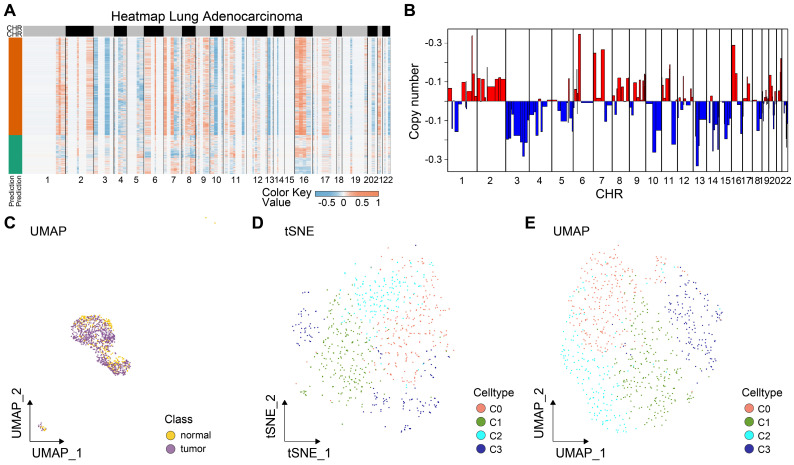
** Reconstructing the pseudotime trajectory of tumor cells using lung epithelial cells and tumor cells and identifying genes varied during the trajectory.** (A) Heatmap of CNV levels in lung epithelial cells. Green represents normal lung epithelial cells, and yellow represents malignant lung epithelial cells (tumor) in legend. Color key from deep blue to yellow indicates relative CNV levels from low to high. (B) The copy number variation of genes at different locations of chromosomes in all tumor cells. (C) Normal and tumor cells of LUAD patients are identified by using the uniform manifold approximation and projection (UMAP). Tumor cell types (D-E) in LUAD samples demonstrated using the uniform manifold approximation and projection (UMAP) and t-distributed stochastic neighbor embedding (TSNE) plots according to their featured gene expression profiles.

**Figure 3:**
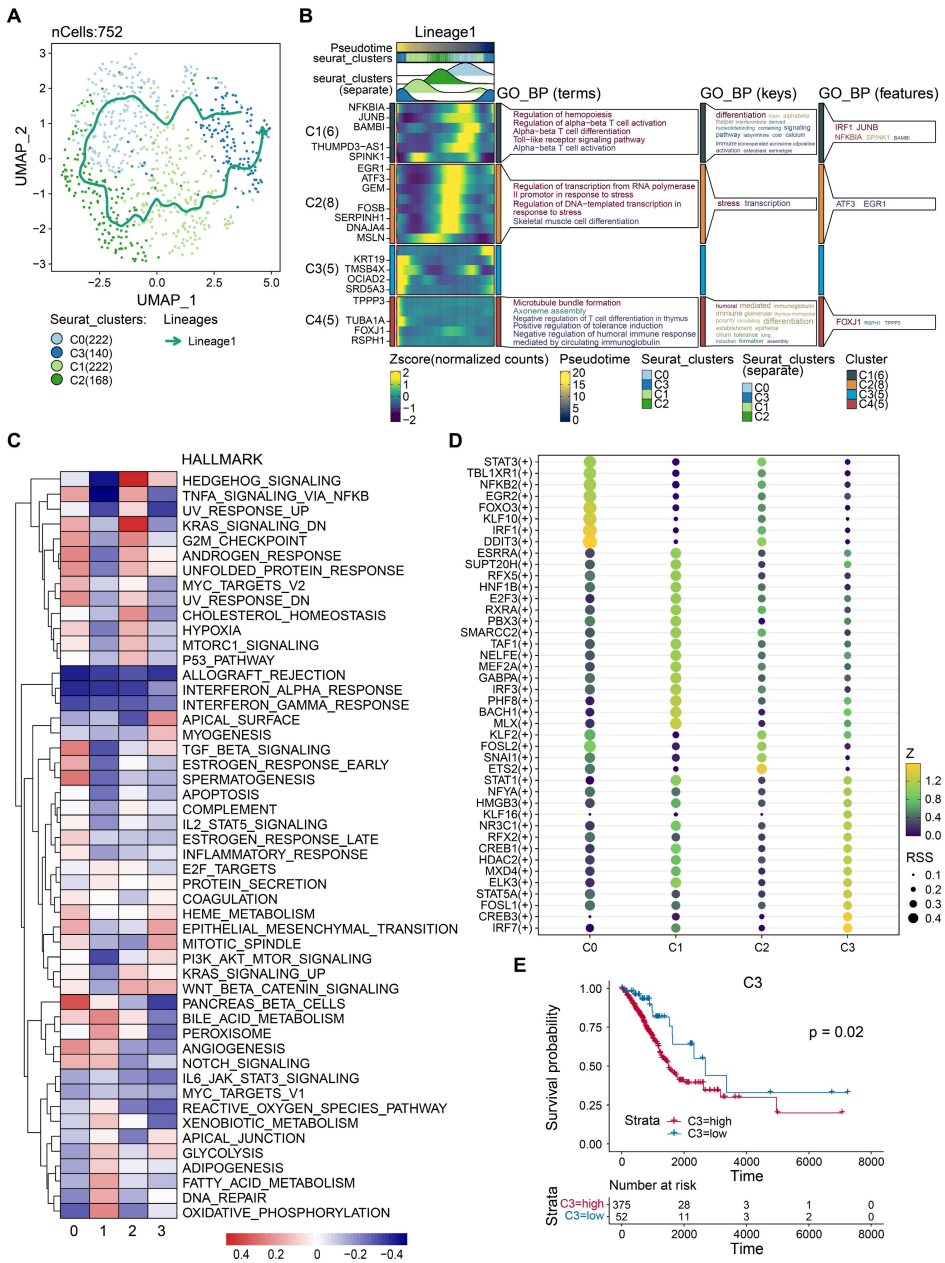
** Identification of critical prognostic genes for LUAD.** (A) Cell type assignment following UMAP-based visualization of expression differences for 11,655 single lung alveolar epithelial cells (normal and malignant lung epithelial cells) from 3 tumor samples in the scRNA-seq dataset. Cell trajectory of tumor cells was generated using the slingshot algorithm in SCP. Lineages represented cell trajectory directions. (B) The differential expressed genes (DEGs) with expression levels that changed the most over the pseudotime trajectory were divided into 4 clusters based on their expression trend, and the representative processes of each cluster are shown. Color key from deep blue to yellow indicates relative expression levels of the DEGs from low to high. (C) Dot plot showing the representative biological processes enriched in each subgroup. (D) Activated transfactors in four different subgroups of tumor cells (E) Kaplan-Meier plot predicting overall survival probability of LUAD patients of C3 group.

Tumor tissues comprise both malignant and benign epithelial cells, and SCEVAN offers promise in distinguishing these cell populations within a tumor microenvironment. As presented in Fig. 2A, the yellow on the left represents the malignant epithelial cells, while the green represents normal epithelial cells. It was obvious that the malignant epithelial cells showed significantly higher levels of amplification and deletion. Furthermore, the CNV of malignant epithelial cells is demonstrated in Fig. 2B. Additionally, the distribution of tumor and normal epithelial cells was visualized by UMAP. We re-clustered the tumor cells into four groups based on PCA to explore the malignant cells further. As displayed in Figs. 2D-E, the four distinct subgroups were separated, indicating the heterogeneity of tumor cells. To unravel the dynamic transition of epithelial cells within the LUAD microenvironment, we employed cell trajectory analysis. This approach allowed us to infer the potential developmental path from normal epithelial cells to tumor cells. As depicted in Figs. 3A and C, the trajectory initiation featured tumor cells from the C0 cluster, while tumor cells from the C3 cluster were situated at the endpoint of the trajectory. To identify key genes and biological processes driving LUAD progression, we investigated the dynamic changes in gene expression patterns within the "lineage 1" trajectory derived from the cell trajectory analysis. As depicted in Fig. 3B, we identified the most significant genes implicated in LUAD progression: *NFKBIA*, *JUNB*, *BAMBI*, *THUMPD3-AS1*, *SPINK1*, *EGR1*, *ATF3*, *GEM*, *FOSB*, *SERPINH1*, *DNAJA4*, MSLN, *KRT19*, *TMSB4X*, *OCIAD2*, *SRD5A3*, *TPPP3*, *TUBA1A*, *FOXJ1*, and *RSPH1*. The biological progress, including regulation of alpha-beta T cell activation and differentiation, Toll-like receptor signaling pathway, regulation of transcription from RNA polymerase II promoter in response to stress, and positive regulation of tolerance induction, were potentially associated with LUAD progression. These observations disclosed the dynamic characteristics of biological processes throughout the trajectory, extending from C0 cluster tumor cells to C3 cluster tumor cells. Moreover, the genes identified in this context, closely associated with tumor cell development, hold the potential to function as valuable biomarkers for monitoring and understanding LUAD progression. To delve deeper into the distinctions among the four subgroups, single-sample gene set enrichment analysis (ssGSEA) was conducted. As indicated in Fig. 3C, tumor cells of the C0 group enriched in TGF-β signaling and angiogenesis, G2M checkpoint, tumor cells of the C2 group enriched in hedgehog signaling, *KRAS* signaling, and G2M checkpoint, tumor cells of the C3 group enriched in epithelial-mesenchymal transition (EMT). Collectively, these results implied that four tumor subgroups exhibited different biological differences. Moreover, transcription factor (TF) is a critical regulator for cell fate. We performed TF analysis for distinct subgroups (Fig. 3D). For instance, the TFs, including *STAT3*, *TBL1XR1*, *NFKB2*, *EGR2*, *FOXO3*, *KLF10*, *IRF1*, and *DDIT3* were more activated in C0 subgroup than other subgroups. TFs including *STAT1*, *NFYA*, *HMGB3*, *KLF16*, *NR3C1*, *RFX2*, *CREB1*, *HDAC2*, *MXD4*, *ELK3*, *STAT5A*, *FOSL1*, *CREB3*, and *IRF7* were more activated in C3 subgroup. These results preliminarily demonstrated the differences in TF activity between subpopulations and revealed the characteristics of each subpopulation. To estimate the prognostic value of the subgroups, we inferred the cell proportions in the TCGA-LUAD dataset by cibersoftX. As presented in Fig. 3E, only the C3 subgroup showed poor prognostic value in early-stage LUAD (Supplementary Figure S1), suggesting that the C3 subgroup may promote tumor progression. In summary, four subpopulations of cells demonstrated a significant degree of intratumoral heterogeneity, and the C3 subgroup was a key subgroup that affected the prognosis of patients with early-stage LUAD. Therefore, it suggested that C3 markers hold promise for improving the prediction of prognosis in early-stage LUAD patients.

### Identification of critical prognostic genes for LUAD

**Figure 4:**
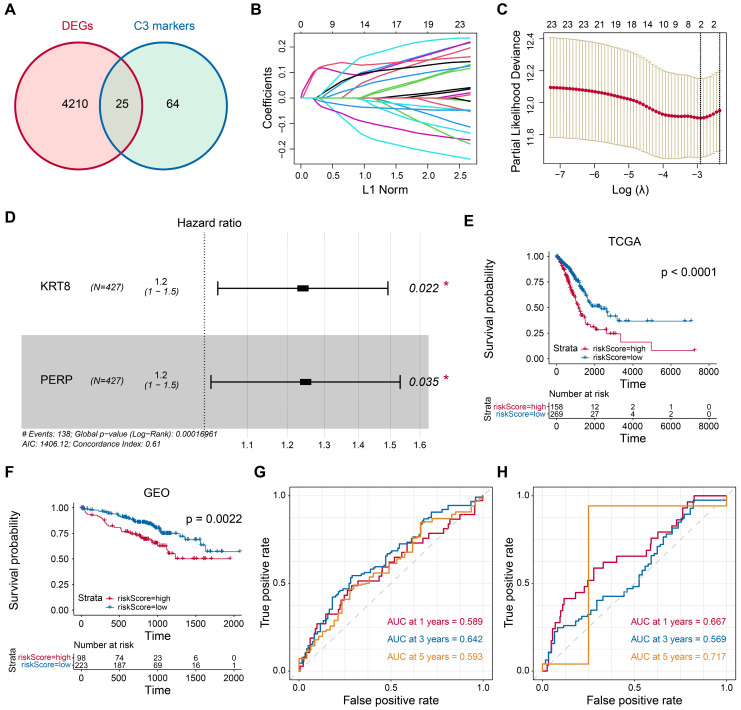
** Identification of candidate prognostic genes.** (A) Venn diagram showing the intersection of DEGs in tumor tissue from the TGCA-LUAD (LUAD) dataset and marker genes of tumor cells from the scRNA-seq dataset. (B) LASSO regression analysis: coefficient values at varying levels of penalty. Each curve represents a gene. (C) Ten-fold cross-validation was used to calculate the best lambda, contributing to the minimum mean cross-validated error. (D) The forest plot derived from multivariable Cox regression analysis reveals two key genes. E-F Patients with tumors were then stratified into high- and low-risk groups based on the optimal cutoff value in the training cohort (E) and validation cohort (F). (G-H) The area under ROC curve was utilized to estimate the discrimination of the model in training set (G) and test set (H).

In the pursuit of identifying crucial prognostic genes for LUAD, a comparative analysis of differential gene expression was conducted by juxtaposing epithelial and tumor cells. Subsequently, we identified 89 markers specific to C3 cluster tumor cells. Through the intersection of these two gene sets, 25 genes were initially pinpointed, as illustrated in Fig. 4A. Further employing the LASSO method, we narrowed down to two critical genes, *KRT8* (HR=1.2, P=0.022) and *PERP* (HR=1.2, P=0.035), as depicted in Figs. 4B-D. Subsequently, based on the optimal cut-off value identified in both the training and validation cohorts (Figs. 4E-F), patients with tumors were categorized into high- and low-risk groups. Patients in the high-risk group showed a poor prognosis. Moreover, time-dependent ROC analysis confirms the robust prognostic value of our model for predicting 1-, 3-, and 5-year OS (Figs. 4G-H). Statistical analyses were conducted to explore the association between transcriptomic profiles and specific clinical parameters, such as survival outcomes.

### Nomogram construction

**Figure 5:**
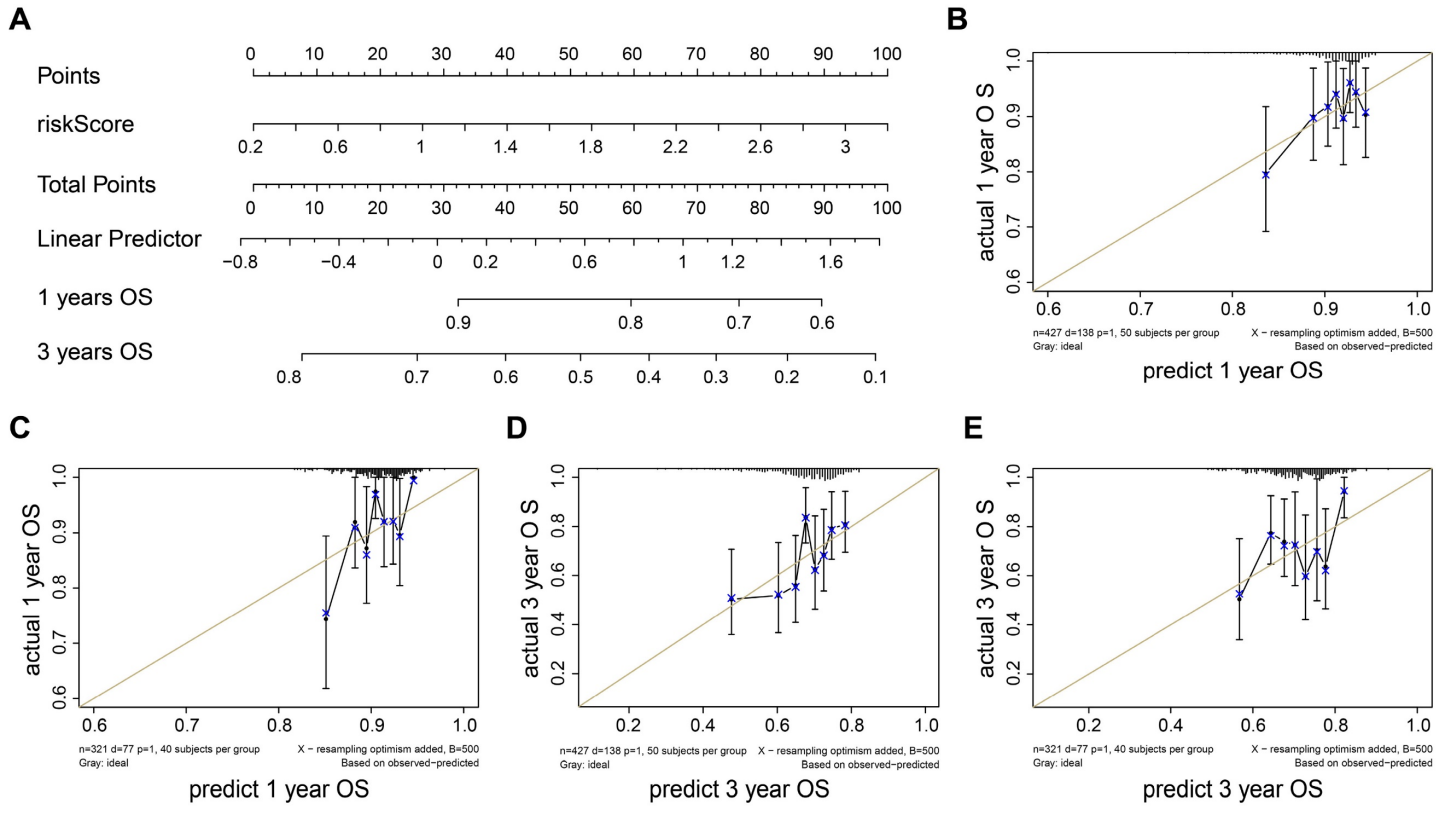
** Validation of a diagnostic model for LUAD.** (A) Nomogram to estimate the risk of LUAD patient. (B-E) Calibrate curves show our model has a prognostic efficiency in predicting 1- (B-C) and 3- (D-E) years OS in training and validation cohort.

**Figure 6:**
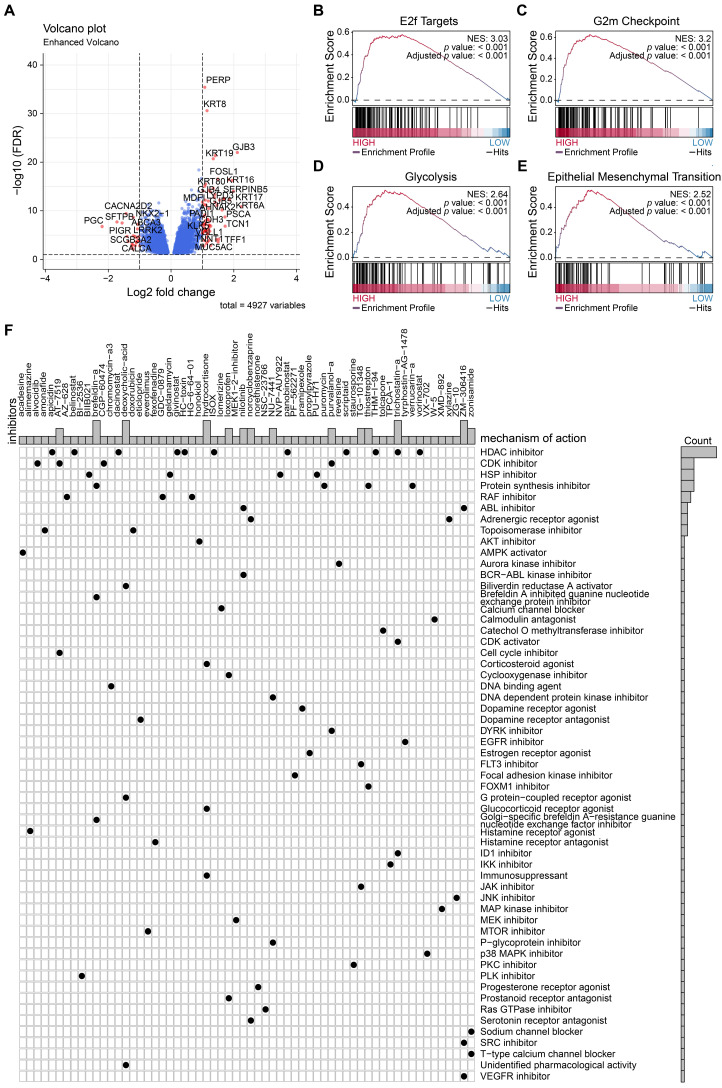
** Potential drugs for high-risk patients.** (A) Differential gene expression between low- and high-risk groups of patients with tumors. (B-E) The GSEA analysis revealed a significant enrichment of the high-risk group in pathways related to E2F targets, G2M checkpoint, glycolysis, and EMT. (F) Candidate drugs for high-risk group patients.

To construct the prognostic nomogram for early-stage LUAD, logistic regression was employed to construct a predictive model for LUAD risk based on the assigned risk scores (Fig. 5A). Calibration curves showed that our model demonstrated high efficiency in predicting 1- and 3-year overall survival (OS) in both the training and validation cohorts. (Figs. 5B-E). Furthermore, we analyzed differential gene expression between low- and high-risk groups of patients with tumors (Fig. 6A). *PERP* and *KRT8* were also upregulated. Then, to elucidate the potential biological functions associated with the 2-mRNA signature, gene set enrichment analysis (GSEA) was performed to explore the crucial pathways associated with the high-risk groups. As depicted in Figs. 6B-E, the GSEA analysis revealed a significant enrichment of the high-risk group in pathways related to E2F targets (B), G2M checkpoint (C), glycolysis (D), and EMT (E). These findings hint at a potential association between the high-risk group and the activation or modulation of specific biological processes. The results further revealed heightened tumor proliferation and aggressiveness in patients belonging to the high-risk group. Given the poor prognosis of the high-risk group, there is a pressing need for additional potential drugs for intervention. We identified several drugs for high-risk group patients (Fig. 6F). For example, AT-7519, a cyclin-dependent kinase (CDK) inhibitor and a cell cycle inhibitor, emerges as a potential candidate drug for patients classified as high risk.

In a word, the nomogram we constructed is a promising tool for predicting early-stage LUAD prognosis.

### KRT8 and PERP expression in patients with LUAD

**Figure 7:**
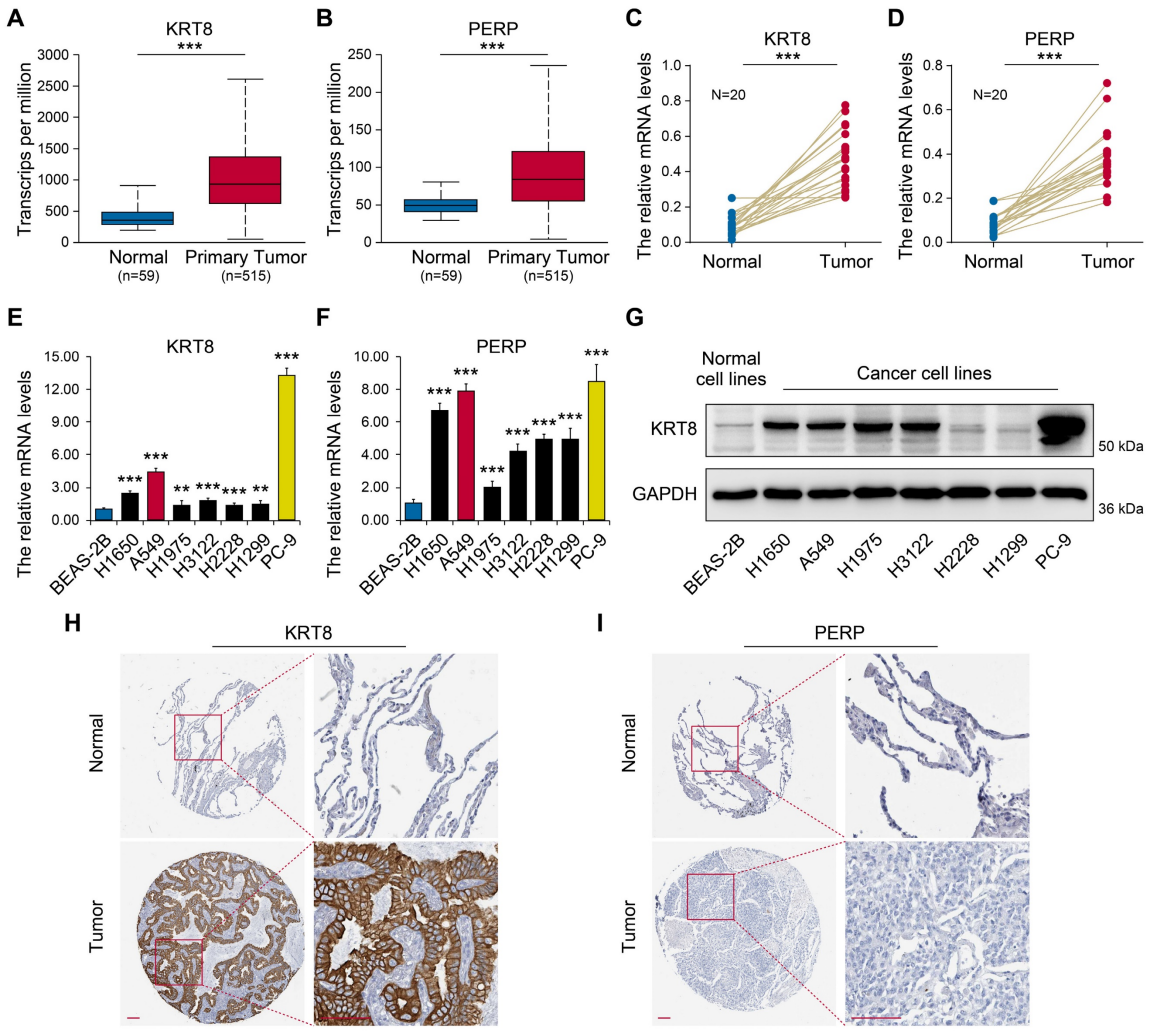
** Expression validation of candidate genes.** (A-B) Transcriptional levels of KRT8 and PERP between LUAD and normal tissues using UALCAN. (C-D) The mRNA levels of KRT8 and PERP in LUAD and the corresponding normal samples from patients were detected by RT-qPCR. (E-F) The levels of KRT8 and PERP in different LUAD cell lines by RT-qPCR. (G) The levels of KRT8 and PERP in different LUAD cell lines by western blot. H-I Immunohistochemistry (IHC) analysis of *KRT8* and *PERP* protein expression in LUAD tissues and corresponding normal controls. The data were retrieved from The Human Protein Atlas (https://www.proteinatlas.org), a publicly available database providing IHC-based expression data.

To investigate the expression patterns of *KRT8* and *PERP* in LUAD, UALCAN was employed (Figs. 7A-B). The results showed significantly elevated *KRT8* and *PERP* expression in LUAD tissues. To validate these findings, we assessed the mRNA levels of *KRT8* and *PERP* in LUAD and corresponding normal samples from patients using RT-qPCR. Consistent with the database, the RT-qPCR results revealed a significant upregulation of *KRT8* and *PERP* in LUAD compared to normal tissues. (Fig 7C-D). Next, we evaluated the expression levels of *KRT8* and *PERP* in various LUAD cell lines using RT-qPCR. (Fig 7E-F) and western blot (Fig. 7G). The results demonstrated high expression levels of *KRT8* and *PERP* in LUAD cell lines, while the western blot showed similar results. To investigate protein expression, we analyzed immunohistochemistry data from The Human Protein Atlas (HPA) for *KRT8* and *PERP* in LUAD tissues and their corresponding normal controls. Our analysis revealed a significant upregulation of *KRT8* protein in LUAD compared to normal tissues (Fig. 7H). However, no difference was observed in the protein levels of *PERP*, possibly attributable to the extremely low mRNA levels of *PERP* in both LUAD and normal tissues (Fig. 7I).

### KRT8 and PERP promote LUAD cell proliferation and migration *in vitro*

**Figure 8:**
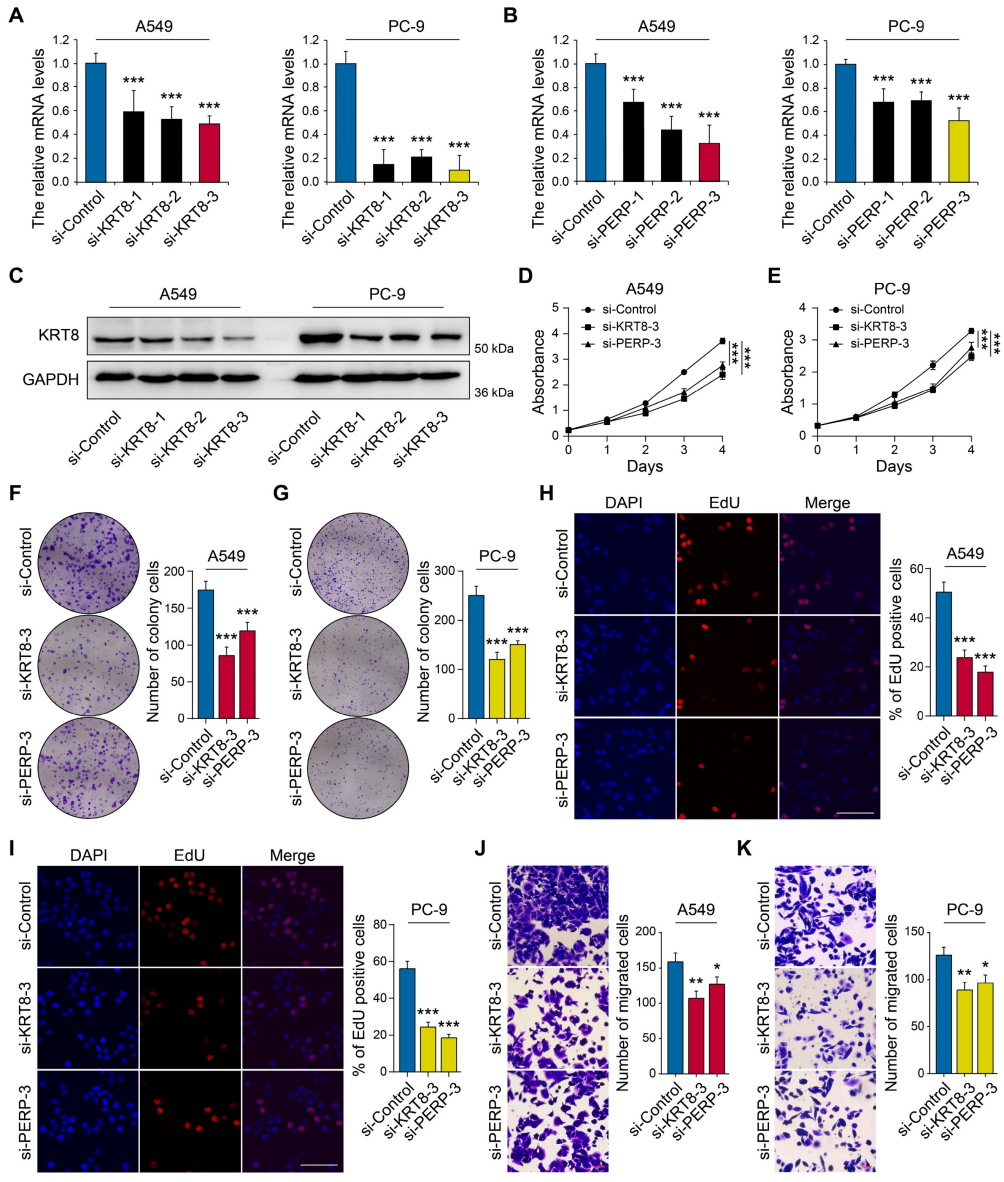
** Knockdown of KRT8 and PERP inhibited LUAD cell proliferation and migration.** (A-B) The mRNA levels of KRT8 and PERP in in KRT8/PERP-knockdown A549 and PC-9 cells and negative control cells. (C) Western blotting analysis of KRT8 expression in KRT8-knockdown A549 and PC-9 cells and negative control cells. (D-E) The CCK8 assays of different LUAD cell lines were analyzed. (F-G) The colony formation assays of different LUAD cell lines were analyzed. (H-I) The EdU analysis of different LUAD cell lines were analyzed. J-K A comparison of migration assays between LUAD cell lines was performed.

To elucidate the functional roles of *KRT8* and *PERP* in LUAD, we next performed *in vitro* experiments to assess their impact on key biological behaviors of LUAD cell lines, such as proliferation and invasion. The RT-qPCR results demonstrated elevated expression of *KRT8* and *PERP* in LUAD cell lines, particularly in A549 and PC-9. Next, we used three specific siRNA targeting *KRT8* and *PERP*, and one of them could reduce the *KRT8* and *PERP* expression in A549 and PC-9 cell lines (Figs. 8A-C). The CCK8 (Figs. 8D-E) and colony formation assays (Figs. 8F-G) and EdU analysis (Figs. 8H-I) indicated that *KRT8* and *PERP* depletion inhibited LUAD cell proliferation, respectively. The transwell assays indicated that the depletion of *KRT8* and *PERP* inhibited LUAD cell migration (Figs. 8J-K). These results suggest that *KRT8* and *PERP* may function as tumor oncogenes in LUAD.

## Discussion

Numerous transcriptomic and genomic investigations have been undertaken to unravel the prognostic outlook of lung cancer and pinpoint potential biomarkers for prognosis^19^-^21^. Therefore, multiple efficacious prognostic markers are now positioned for incorporation into the clinical management of lung cancer. These strides hold the potential to augment our capability to foresee outcomes and customize more individualized and effective therapeutic strategies for individuals with lung cancer.^22^, ^23^. Nonetheless, given the highly heterogeneous nature of LUAD, the predominant type of lung cancer, the challenge lies in comprehensively understanding and addressing its diversity.

Previous studies have predominantly relied on transcriptomics to investigate RNA expression in tumor tissues, but they may not accurately capture variations in gene expression among distinct cells within the tissue. The advent of single-cell sequencing has provided a breakthrough in discerning changes in gene expression at the cellular level^24^-^26^. Thus, leveraging advancements in sequencing technology enables a more precise characterization of the TME in LUAD. This approach holds promise for improved clinical outcomes in cancer patients by identifying specific tumor biomarkers, it allows for more accurate diagnosis and the development of individualized treatment plans, potentially leading to better treatment efficacy^27^-^29^.

This study employed a comprehensive approach to analyze the TME of LUAD patients. We successfully identified lung tumor cells and explored potential trajectories involving the differentiation of lung epithelial cells into tumor cells through CNV analysis. Our analysis identified several genes closely associated with tumor cell differentiation: *NFKBIA*, *JUNB*, *BAMBI*, *THUMPD3-AS1*, *SPINK1*, *EGR1*, *ATF3*, *GEM*, *FOSB*, *SERPINH1*, *DNAJA4*, *MSLN*, *KRT19*, *TMSB4X*, *OCIAD2*, *SRD5A3*, *TPPP3*, *TUBA1A*, *FOXJ1*, and *RSPH1*. Previous researches have explored the mechanism of action and prognostic value of *JUNB*^30^, *BAMBI*^31^, *ATF3*^32^, *MSLN*^33^, *KRT19*^34^, *SPINK1*^35^ and *EGR1*^36^ in NSCLC. This suggests that the genes identified through our cell trajectory analysis might play a crucial role in driving LUAD development. However, to validate their functional significance, further *in vitro* and *in vivo* studies are warranted.

Upon further subdivision of the tumor cells, we successfully identified four distinct tumor subgroups. The involvement of these tumor subgroups in distinct biological processes highlights a substantial degree of heterogeneity among them. This diversity underscores the complexity and varied nature of biological processes within the context of the identified tumor subgroups.

The C0 subgroup of tumor cells identified by us is upregulated in the *TGF-β* signal pathway, which indicates that it may be related to the early immune response of the tumor. In the initial phases of tumors, *TGF-β* is acknowledged as a tumor suppressor, impeding proliferation and triggering apoptosis^37^. *IRF1* was significantly activated in C0 tumor cell subsets. A transcriptional regulator and tumor suppressor, the protein encoded by the *IRF1* gene activates genes associated with both innate and acquired immune responses^38^, ^39^. As a tumor suppressor, it can inhibit tumor cell growth and stimulate immune response against these cells^40^. In our study, high levels of IRF1 activation may be associated with an early tumor immune response. Max-like protein X (*MLX*) was significantly activated in C1 tumor cell subsets. Heterodimers with Mad proteins are formed by the gene products of *MLX*, and they play a role in proliferation, determination, and differentiation. This may be related to the active proliferation of tumor cells. A research suggests an association between *MLX* and the initiation and progression of hepatocellular carcinoma^41^. The C2 subgroup of tumor cells we identified is upregulated in *KRAS* signaling pathway. Researches disclosed that when the carcinogenic *KRAS* signaling pathway is activated, it promotes inhibitory cytokines and chemokines secretion in alveolar cells, thus affecting the recruitment and polarization of various immune cells, shaping the inhibitory TME, which is conducive to the tumor development^42^. *ETS2* was significantly activated in C2 tumor cell subsets. *ETS2* is a transcription factor and proto-oncogene that regulates development, apoptosis, and telomerase.

The oncogenic role of *ETS2* is substantiated by evidence in bladder cancer^43^. There is also evidence suggesting that *ETS2* mediates the formation of osteosarcoma by GOF (gain-of-function) mutants of *p53*, leading to increased lung metastatic ability^44^. This observation suggests a close correlation between the onset and progression of the disease in patients with early-stage tumors. The C3 subgroup of tumor cells we identified is upregulated in the EMT pathway, which may indicate the tendency of cell proliferation, differentiation, and metastasis. Moreover, we found that only the C3 group displayed a marked association with poor prognosis in early-stage LUAD patients. Therefore, further exploring C3 markers may help us find therapeutic targets for patients with early LUAD.

We crossed genes upregulated in tumor cells to identify effective prognostic markers with those upregulated in LUAD tissue. Identified as prognostic biomarkers for LUAD, *KRT8* and *PERP* underwent follow-up experiments for verification. Gene expression data confirms the accuracy of our analysis. Although additional research and clinical trials are imperative for confirmation, published studies support the prognostic potential of these two genes for LUAD.

For example, *KRT8* is a notable member belonging to the keratin family, and its products usually dimerize with *KRT18* to form intermediate filaments in simple monolayer epithelial cells, which is vital for maintaining cell structural integrity, signal transduction, and cell differentiation. A study identified *KRT8* as a potential novel biomarker for LUAD, showcasing its role in fostering metastasis and triggering EMT through *NF-κB* signaling^45^. Previous research has established a link between high *KRT8* expression and a poor prognosis for LUAD patients^46^. Integrating our findings, we observed that knocking down *KRT8* in A549 and PC9 cell lines resulted in the suppression of tumor cell proliferation and invasion. This also preliminarily confirms the accuracy of our bioinformatics analysis.

The tetraspan plasma membrane protein *PERP* (*p53* apoptosis effector related to *PMP22*) is a lesser-known transcriptional target of *p53* and *p63*^47^. PERP is highly expressed in lung cancer. Stable knockdown of *PERP* expression induced death of CL1-5 and A549 lung cancer cells^48^. However, the *PERP* expression in our experiments is completely different at the RNA and protein levels, which warrants further investigation. The high-risk group of patients with LUAD was significantly enriched in the E2F targets, G2M checkpoint, glycolysis, and the EMT. All of these factors are intricately linked to the proliferation and metastasis of tumor cells, and we have screened some drug candidates for these properties. For example, alvocidib and purvalanol-a may function as a CDK inhibitor to inhibit tumor proliferation and metastasis.

Our findings highlight the potential of these candidate genes for both predicting patient prognosis and accurately discriminating between benign and malignant lung tissues. Moreover, the predictive model we developed demonstrates robust differentiation and accuracy, as evidenced by its performance on the training and the external test sets, indicative of its stability and practical utility. Compared to previous studies, such as Yang *et al.*^49^, which focused on fibroblasts and their impact on LUAD progression, our study uniquely integrates single-cell and bulk transcriptomic analyses to explore epithelial cell transformation and tumor heterogeneity. Furthermore, while studies like Xiao *et al.*^50^ and Kim *et al.*^51^ identified circulating biomarkers, we focused on LUAD tissue-specific markers and their functional validation, offering new insights into tumor biology and potential clinical applications. Nevertheless, certain limitations in our study should be acknowledged. This study utilized a relatively small sample size of 20 paired lung cancer and adjacent non-cancerous tissues for downstream validations, including RT-qPCR and immunohistochemistry. While these results provide initial insights, we recognize that the statistical power of these analyses is limited. Future studies will involve a larger cohort to validate and strengthen the robustness of these findings. Moreover, the availability of RNA-seq data was limited. To unveil the broader applicability of microenvironmental features in LUAD, it is imperative to gather additional single-cell data. Finally, although we examined and validated six potential biomarkers at the mRNA level, their practical use requires further experimental and clinical studies.

In summary, our study aimed to characterize the TME of LUAD. Our comprehensive exploration of the heterogeneity among lung tumor cells is noteworthy. Additionally, we have successfully developed a prognostic model grounded in two candidate prognostic markers for LUAD. Our findings shed light on LUAD heterogeneity and offer a theoretical basis for prognostic stratification in LUAD.

## Supplementary Material

Supplementary figure and tables.

## Abbreviations

LUAD: Lung Adenocarcinoma
TCGA: The Cancer Genome Atlas
PCA: Principal Component Analysis
GEO: Gene Expression Omnibus
ScRNA: Single-cell RNA
EMT: Epithelial-mesenchymal Transition
GO: Gene Ontology
KEGG: Kyoto Encyclopedia of Genes and Genomes
GSEA: Gene Set Enrichment Analysis
NT: Normal Tissue
TME: Tumor Microenvironment
RT-qPCR: Reverse Transcription-Quantitative Polymerase Chain Reaction
CDK: Cyclin-Dependent Kinase

## References

[1] Bray F, Ferlay J, Soerjomataram I, Siegel RL, Torre LA, Jemal A (2018). Global cancer statistics 2018: GLOBOCAN estimates of incidence and mortality worldwide for 36 cancers in 185 countries. CA Cancer J Clin.

[2] Thai AA, Solomon BJ, Sequist LV, Gainor JF, Heist RS (2021). Lung cancer. Lancet.

[3] Lovly CM (2022). Expanding Horizons for Treatment of Early-Stage Lung Cancer. N Engl J Med.

[4] Goldstraw P, Ball D, Jett JR, Le Chevalier T, Lim E, Nicholson AG (2011). Non-small-cell lung cancer. Lancet.

[5] Neal RD, Sun F, Emery JD, Callister ME (2019). Lung cancer. BMJ.

[6] Gridelli C, Rossi A, Carbone DP, Guarize J, Karachaliou N, Mok T (2015). Non-small-cell lung cancer. Nat Rev Dis Primers.

[7] Elhanani O, Ben-Uri R, Keren L (2023). Spatial profiling technologies illuminate the tumor microenvironment. Cancer Cell.

[8] Zhao W, Zhu B, Hutchinson A, Pesatori AC, Consonni D, Caporaso NE (2022). Clinical Implications of Inter- and Intratumor Heterogeneity of Immune Cell Markers in Lung Cancer. J Natl Cancer Inst.

[9] Ko KP, Huang Y, Zhang S, Zou G, Kim B, Zhang J (2023). Key Genetic Determinants Driving Esophageal Squamous Cell Carcinoma Initiation and Immune Evasion. Gastroenterology.

[10] Zou G, Huang Y, Zhang S, Ko KP, Kim B, Zhang J (2024). E-cadherin loss drives diffuse-type gastric tumorigenesis via EZH2-mediated reprogramming. J Exp Med.

[11] Tian Y, Li Q, Yang Z, Zhang S, Xu J, Wang Z (2022). Single-cell transcriptomic profiling reveals the tumor heterogeneity of small-cell lung cancer. Signal Transduct Target Ther.

[12] Kim B, Zhang S, Huang Y, Ko KP, Jung YS, Jang J (2024). CRACD loss induces neuroendocrine cell plasticity of lung adenocarcinoma. Cell Rep.

[13] Wang M, Herbst RS, Boshoff C (2021). Toward personalized treatment approaches for non-small-cell lung cancer. Nat Med.

[14] Chandrashekar DS, Bashel B, Balasubramanya SAH, Creighton CJ, Ponce-Rodriguez I, Chakravarthi B (2017). UALCAN: A Portal for Facilitating Tumor Subgroup Gene Expression and Survival Analyses. Neoplasia.

[15] Asplund A, Edqvist PH, Schwenk JM, Ponten F (2012). Antibodies for profiling the human proteome-The Human Protein Atlas as a resource for cancer research. Proteomics.

[16] Tian Y, Yu Y, Hou LK, Chi JR, Mao JF, Xia L (2016). Serum deprivation response inhibits breast cancer progression by blocking transforming growth factor-beta signaling. Cancer Sci.

[17] Zhu K, Wang B, Li Y, Yu Y, Chen Z, Yue H (2023). CAVIN2/SDPR Functioned as a Tumor Suppressor in Lung Adenocarcinoma from Systematic Analysis of Caveolae-Related Genes and Experimental Validation. J Cancer.

[18] Li Y, Wu J, Tian Y, Zhu Q, Ge Y, Yu H (2022). MED1 Downregulation Contributes to TGFbeta-Induced Metastasis by Inhibiting SMAD2 Ubiquitination Degradation in Cutaneous Melanoma. J Invest Dermatol.

[19] Li MY, Liu LZ, Dong M (2021). Progress on pivotal role and application of exosome in lung cancer carcinogenesis, diagnosis, therapy and prognosis. Mol Cancer.

[20] Tang H, Wang S, Xiao G, Schiller J, Papadimitrakopoulou V, Minna J (2017). Comprehensive evaluation of published gene expression prognostic signatures for biomarker-based lung cancer clinical studies. Ann Oncol.

[21] Goyal B, Yadav SRM, Awasthee N, Gupta S, Kunnumakkara AB, Gupta SC (2021). Diagnostic, prognostic, and therapeutic significance of long non-coding RNA MALAT1 in cancer. Biochim Biophys Acta Rev Cancer.

[22] Zhang C, Wang H (2022). Accurate treatment of small cell lung cancer: Current progress, new challenges and expectations. Biochim Biophys Acta Rev Cancer.

[23] Wood SL, Pernemalm M, Crosbie PA, Whetton AD (2015). Molecular histology of lung cancer: from targets to treatments. Cancer Treat Rev.

[24] Lei Y, Tang R, Xu J, Wang W, Zhang B, Liu J (2021). Applications of single-cell sequencing in cancer research: progress and perspectives. J Hematol Oncol.

[25] Li PH, Kong XY, He YZ, Liu Y, Peng X, Li ZH (2022). Recent developments in application of single-cell RNA sequencing in the tumour immune microenvironment and cancer therapy. Mil Med Res.

[26] Hwang B, Lee JH, Bang D (2018). Single-cell RNA sequencing technologies and bioinformatics pipelines. Exp Mol Med.

[27] Van de Sande B, Lee JS, Mutasa-Gottgens E, Naughton B, Bacon W, Manning J (2023). Applications of single-cell RNA sequencing in drug discovery and development. Nat Rev Drug Discov.

[28] Suva ML, Tirosh I (2019). Single-Cell RNA Sequencing in Cancer: Lessons Learned and Emerging Challenges. Mol Cell.

[29] Hedlund E, Deng Q (2018). Single-cell RNA sequencing: Technical advancements and biological applications. Mol Aspects Med.

[30] Suphakhong K, Terashima M, Wanna-Udom S, Takatsuka R, Ishimura A, Takino T (2022). m6A RNA methylation regulates the transcription factors JUN and JUNB in TGF-beta-induced epithelial-mesenchymal transition of lung cancer cells. J Biol Chem.

[31] Marwitz S, Depner S, Dvornikov D, Merkle R, Szczygiel M, Muller-Decker K (2016). Downregulation of the TGFbeta Pseudoreceptor BAMBI in Non-Small Cell Lung Cancer Enhances TGFbeta Signaling and Invasion. Cancer Res.

[32] Ku HC, Cheng CF (2020). Master Regulator Activating Transcription Factor 3 (ATF3) in Metabolic Homeostasis and Cancer. Front Endocrinol (Lausanne).

[33] Morello A, Sadelain M, Adusumilli PS (2016). Mesothelin-Targeted CARs: Driving T Cells to Solid Tumors. Cancer Discov.

[34] Mehrpouya M, Pourhashem Z, Yardehnavi N, Oladnabi M (2019). Evaluation of cytokeratin 19 as a prognostic tumoral and metastatic marker with focus on improved detection methods. J Cell Physiol.

[35] Tiwari R, Pandey SK, Goel S, Bhatia V, Shukla S, Jing X (2015). SPINK1 promotes colorectal cancer progression by downregulating Metallothioneins expression. Oncogenesis.

[36] Ferraro B, Bepler G, Sharma S, Cantor A, Haura EB (2005). EGR1 predicts PTEN and survival in patients with non-small-cell lung cancer. J Clin Oncol.

[37] Moon H, Han KH, Ro SW (2017). Pro-tumorigenic roles of TGF-beta signaling during the early stages of liver tumorigenesis through upregulation of Snail. BMB Rep.

[38] Zhou H, Tang YD, Zheng C (2022). Revisiting IRF1-mediated antiviral innate immunity. Cytokine Growth Factor Rev.

[39] Feng H, Zhang YB, Gui JF, Lemon SM, Yamane D (2021). Interferon regulatory factor 1 (IRF1) and anti-pathogen innate immune responses. PLoS Pathog.

[40] Tanaka N, Taniguchi T (2000). The interferon regulatory factors and oncogenesis. Semin Cancer Biol.

[41] Yu A, Yu P, Zhu Y, Zhu R, Sun R, Ye D (2023). Glucose-induced and ChREBP: MLX-mediated lipogenic program promotes hepatocellular carcinoma development. Oncogene.

[42] Mugarza E, van Maldegem F, Boumelha J, Moore C, Rana S, Llorian Sopena M (2022). Therapeutic KRAS(G12C) inhibition drives effective interferon-mediated antitumor immunity in immunogenic lung cancers. Sci Adv.

[43] Zhu J, Xu C, Ruan L, Wu J, Li Y, Zhang X (2019). MicroRNA-146b Overexpression Promotes Human Bladder Cancer Invasion via Enhancing ETS2-Mediated mmp2 mRNA Transcription. Mol Ther Nucleic Acids.

[44] Liu DD, Kang Y (2017). Ets2 anchors the prometastatic function of mutant p53 in osteosarcoma. Genes Dev.

[45] Chen H, Chen X, Pan B, Zheng C, Hong L, Han W (2022). KRT8 Serves as a Novel Biomarker for LUAD and Promotes Metastasis and EMT via NF-kappaB Signaling. Front Oncol.

[46] Xie L, Dang Y, Guo J, Sun X, Xie T, Zhang L (2019). High KRT8 Expression Independently Predicts Poor Prognosis for Lung Adenocarcinoma Patients. Genes (Basel).

[47] Roberts O, Paraoan L (2020). PERP-ing into diverse mechanisms of cancer pathogenesis: Regulation and role of the p53/p63 effector PERP. Biochim Biophys Acta Rev Cancer.

[48] Liao CY, Yang SF, Wu TJ, Chang H, Huang CF, Liu YF (2021). Novel function of PERP-428 variants impacts lung cancer risk through the differential regulation of PTEN/MDM2/p53-mediated antioxidant activity. Free Radic Biol Med.

[49] Yang H, Sun B, Fan L, Ma W, Xu K, Hall SRR (2022). Multi-scale integrative analyses identify THBS2(+) cancer-associated fibroblasts as a key orchestrator promoting aggressiveness in early-stage lung adenocarcinoma. Theranostics.

[50] Xiao Y, Hu F, Chi Q (2024). Single-cell RNA sequencing and spatial transcriptome reveal potential molecular mechanisms of lung cancer brain metastasis. Int Immunopharmacol.

[51] Kim J, Xu Z, Marignani PA (2021). Single-cell RNA sequencing for the identification of early-stage lung cancer biomarkers from circulating blood. NPJ Genom Med.

