# Supplementary material for: Integration of Single-Cell and Bulk Transcriptomes to Identify a Poor Prognostic Tumor Subgroup to Predict the Prognosis of Patients with Early-stage Lung Adenocarcinoma
Source: J Cancer. 2025 Jan 21;16(4):1397–412. doi: 10.7150/jca.105926 (PMC11786047; doi:10.7150/jca.105926)
Supplement: Supplementary file 1 — Supplementary figure and tables. [file jcav16p1397s1.pdf]

Supplementary Figure

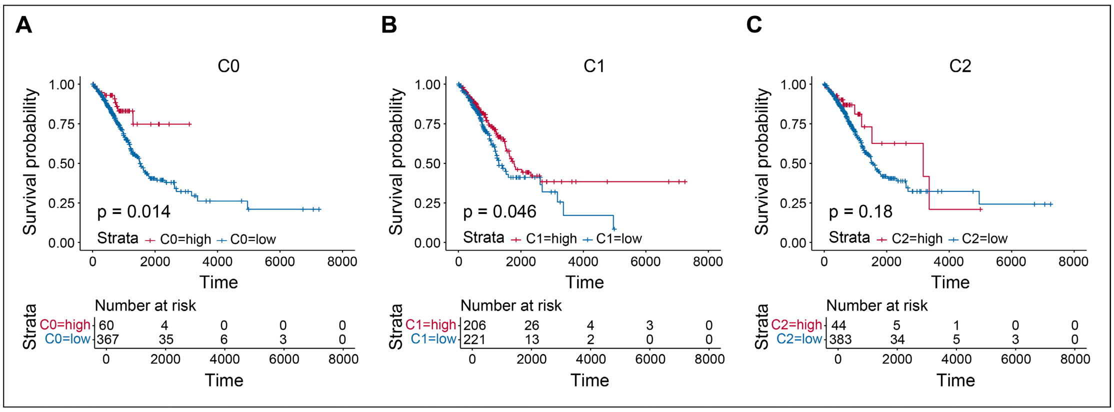

Figure S1. The prognostic value of distinct cell types. **A-C** Kaplan-Meier plot predicting overall survival probability of LUAD patients of C0 (**A**), C1 (**B**), C2 (**C**) group.

Additional File 1

Table S1. Clinicopathological parameters of patients`

	stage	OS	OS.time	cohort
1	I	0	567	TCGA
2	I	0	435	TCGA
3	I	0	2676	TCGA
4	II	1	154	TCGA
5	II	1	995	TCGA
6	I	0	1125	TCGA
7	II	0	813	TCGA
8	I	0	476	TCGA
9	I	0	1285	TCGA
10	I	0	50	TCGA
11	I	0	912	TCGA
12	II	1	87	TCGA
13	II	0	2065	TCGA
14	I	1	3361	TCGA
15	I	0	568	TCGA
16	I	0	520	TCGA
17	I	0	1351	TCGA
18	I	1	987	TCGA
19	II	1	99	TCGA
20	II	1	1516	TCGA
21	I	0	256	TCGA
22	II	1	281	TCGA
23	I	0	2360	TCGA
24	I	0	14	TCGA
25	I	0	2832	TCGA
26	I	0	1400	TCGA
27	I	0	526	TCGA
28	I	0	761	TCGA
29	II	1	1046	TCGA
30	II	1	582	TCGA

31	I	1	469	TCGA
32	I	0	1700	TCGA
33	I	1	1357	TCGA
34	I	0	133	TCGA
35	I	1	949	TCGA
36	I	0	129	TCGA
37	I	1	950	TCGA
38	II	0	630	TCGA
39	II	1	929	TCGA
40	I	0	365	TCGA
41	II	1	116	TCGA
42	I	1	561	TCGA
43	II	0	416	TCGA
44	II	0	701	TCGA
45	I	0	947	TCGA
46	I	0	824	TCGA
47	I	0	484	TCGA
48	I	0	705	TCGA
49	I	0	705	TCGA
50	I	1	1454	TCGA
51	II	1	118	TCGA
52	I	0	418	TCGA
53	II	0	1893	TCGA
54	I	0	2368	TCGA
55	I	1	321	TCGA
56	I	0	2616	TCGA
57	I	1	3169	TCGA
58	I	1	1600	TCGA
59	II	0	1097	TCGA
60	I	0	174	TCGA
61	I	1	1531	TCGA
62	I	1	97	TCGA
63	I	1	97	TCGA
64	I	0	624	TCGA
65	I	0	2248	TCGA

66	I	0	564	TCGA
67	I	1	702	TCGA
68	II	0	1157	TCGA
69	I	0	385	TCGA
70	I	0	323	TCGA
71	I	1	18	TCGA
72	I	1	2620	TCGA
73	I	0	513	TCGA
74	II	1	737	TCGA
75	I	0	351	TCGA
76	I	0	385	TCGA
77	I	0	740	TCGA
78	I	0	2590	TCGA
79	I	1	1197	TCGA
80	I	0	938	TCGA
81	I	0	3305	TCGA
82	I	0	601	TCGA
83	I	0	3261	TCGA
84	II	1	258	TCGA
85	I	0	310	TCGA
86	II	0	426	TCGA
87	I	0	704	TCGA
88	I	0	578	TCGA
89	I	0	862	TCGA
90	I	0	863	TCGA
91	I	0	607	TCGA
92	I	0	3059	TCGA
93	II	0	467	TCGA
94	I	0	1932	TCGA
95	I	0	1324	TCGA
96	I	0	3094	TCGA
97	I	0	2224	TCGA
98	I	0	1617	TCGA
99	I	0	481	TCGA
100	I	0	889	TCGA

101	I	0	1118	TCGA
102	I	0	634	TCGA
103	I	1	1622	TCGA
104	II	1	2027	TCGA
105	I	0	977	TCGA
106	I	0	84	TCGA
107	I	0	466	TCGA
108	I	0	545	TCGA
109	II	1	677	TCGA
110	I	1	503	TCGA
111	I	1	869	TCGA
112	I	0	4992	TCGA
113	I	0	605	TCGA
114	I	0	415	TCGA
115	I	0	377	TCGA
116	II	1	376	TCGA
117	II	0	1119	TCGA
118	II	1	321	TCGA
119	II	0	913	TCGA
120	I	1	244	TCGA
121	I	0	478	TCGA
122	II	0	761	TCGA
123	II	1	1499	TCGA
124	II	0	2823	TCGA
125	II	1	260	TCGA
126	I	0	691	TCGA
127	I	0	824	TCGA
128	II	0	866	TCGA
129	I	0	547	TCGA
130	I	1	777	TCGA
131	II	0	603	TCGA
132	II	1	1501	TCGA
133	I	1	339	TCGA
134	I	1	557	TCGA
135	I	1	4961	TCGA

136	I	1	409	TCGA
137	I	0	600	TCGA
138	I	1	800	TCGA
139	I	0	578	TCGA
140	II	1	896	TCGA
141	I	0	417	TCGA
142	II	0	285	TCGA
143	I	0	534	TCGA
144	I	0	1079	TCGA
145	II	1	343	TCGA
146	I	0	1183	TCGA
147	I	0	1183	TCGA
148	II	1	1778	TCGA
149	II	1	1235	TCGA
150	II	1	237	TCGA
151	I	0	683	TCGA
152	I	0	131	TCGA
153	I	1	550	TCGA
154	I	0	552	TCGA
155	I	0	1333	TCGA
156	I	1	2681	TCGA
157	II	0	84	TCGA
158	I	0	515	TCGA
159	I	0	6732	TCGA
160	I	0	988	TCGA
161	I	1	193	TCGA
162	I	1	119	TCGA
163	II	0	1301	TCGA
164	II	0	1301	TCGA
165	II	1	864	TCGA
166	I	0	842	TCGA
167	I	0	537	TCGA
168	I	0	904	TCGA
169	II	0	492	TCGA
170	I	0	353	TCGA

171	II	0	882	TCGA
172	I	0	610	TCGA
173	II	0	664	TCGA
174	II	1	626	TCGA
175	II	0	60	TCGA
176	I	0	307	TCGA
177	I	0	44	TCGA
178	II	0	553	TCGA
179	I	0	1126	TCGA
180	I	0	423	TCGA
181	I	0	1479	TCGA
182	II	0	422	TCGA
183	I	0	603	TCGA
184	II	1	999	TCGA
185	I	0	573	TCGA
186	I	0	2109	TCGA
187	II	1	2617	TCGA
188	I	1	375	TCGA
189	II	0	11	TCGA
190	I	1	434	TCGA
191	II	0	7062	TCGA
192	II	0	134	TCGA
193	I	0	3759	TCGA
194	II	0	1148	TCGA
195	I	0	872	TCGA
196	II	0	1040	TCGA
197	I	1	58	TCGA
198	I	1	1258	TCGA
199	I	1	468	TCGA
200	II	1	500	TCGA
201	II	1	340	TCGA
202	I	0	487	TCGA
203	I	1	1421	TCGA
204	I	0	62	TCGA
205	I	0	1523	TCGA

206	I	0	718	TCGA
207	I	0	2449	TCGA
208	II	1	760	TCGA
209	II	0	617	TCGA
210	I	0	442	TCGA
211	II	1	124	TCGA
212	I	0	1429	TCGA
213	I	0	1429	TCGA
214	II	0	910	TCGA
215	I	0	476	TCGA
216	I	1	167	TCGA
217	I	0	614	TCGA
218	I	1	1293	TCGA
219	I	0	414	TCGA
220	I	0	730	TCGA
221	I	0	435	TCGA
222	I	0	704	TCGA
223	I	0	415	TCGA
224	II	1	1147	TCGA
225	I	1	1528	TCGA
226	II	0	599	TCGA
227	I	0	531	TCGA
228	I	0	477	TCGA
229	I	0	540	TCGA
230	I	0	568	TCGA
231	I	0	1728	TCGA
232	II	0	677	TCGA
233	II	1	1492	TCGA
234	I	1	308	TCGA
235	I	0	1280	TCGA
236	I	0	1280	TCGA
237	I	1	440	TCGA
238	II	0	1683	TCGA
239	I	1	761	TCGA
240	I	1	761	TCGA

241	I	0	499	TCGA
242	II	1	176	TCGA
243	II	1	1830	TCGA
244	I	0	546	TCGA
245	II	0	1367	TCGA
246	I	1	354	TCGA
247	II	1	336	TCGA
248	II	0	202	TCGA
249	I	0	829	TCGA
250	II	1	442	TCGA
251	I	0	896	TCGA
252	II	0	2067	TCGA
253	I	0	372	TCGA
254	I	1	1135	TCGA
255	II	0	728	TCGA
256	II	0	728	TCGA
257	I	1	434	TCGA
258	I	0	1163	TCGA
259	I	0	629	TCGA
260	I	0	670	TCGA
261	I	1	701	TCGA
262	I	0	7248	TCGA
263	I	0	1126	TCGA
264	I	1	855	TCGA
265	I	0	1036	TCGA
266	I	0	1036	TCGA
267	I	0	2595	TCGA
268	I	0	1369	TCGA
269	I	0	435	TCGA
270	I	1	694	TCGA
271	II	0	806	TCGA
272	I	1	607	TCGA
273	I	0	1750	TCGA
274	II	1	488	TCGA
275	II	0	724	TCGA

276	I	0	225	TCGA
277	I	1	656	TCGA
278	I	1	1215	TCGA
279	II	1	171	TCGA
280	I	0	830	TCGA
281	I	0	462	TCGA
282	I	1	1725	TCGA
283	I	0	705	TCGA
284	I	0	578	TCGA
285	II	0	13	TCGA
286	II	1	586	TCGA
287	I	0	2161	TCGA
288	II	0	791	TCGA
289	II	0	689	TCGA
290	I	0	1175	TCGA
291	II	1	731	TCGA
292	II	0	882	TCGA
293	II	1	428	TCGA
294	II	1	896	TCGA
295	I	0	2199	TCGA
296	I	0	993	TCGA
297	II	0	791	TCGA
298	I	0	141	TCGA
299	II	1	164	TCGA
300	I	0	2973	TCGA
301	I	0	1432	TCGA
302	I	0	888	TCGA
303	II	0	151	TCGA
304	II	0	827	TCGA
305	I	0	889	TCGA
306	II	1	574	TCGA
307	I	1	808	TCGA
308	I	1	808	TCGA
309	II	0	670	TCGA
310	II	0	24	TCGA

311	I	1	995	TCGA
312	II	0	44	TCGA
313	I	0	997	TCGA
314	I	1	1043	TCGA
315	I	0	536	TCGA
316	I	0	260	TCGA
317	I	0	15	TCGA
318	I	1	1268	TCGA
319	I	0	4765	TCGA
320	I	1	1194	TCGA
321	II	1	1209	TCGA
322	I	0	773	TCGA
323	I	0	551	TCGA
324	I	0	652	TCGA
325	I	0	852	TCGA
326	I	0	852	TCGA
327	I	0	1442	TCGA
328	I	0	690	TCGA
329	I	1	653	TCGA
330	I	0	230	TCGA
331	II	1	19	TCGA
332	II	1	291	TCGA
333	II	1	666	TCGA
334	I	0	411	TCGA
335	I	0	592	TCGA
336	I	1	598	TCGA
337	I	0	1189	TCGA
338	I	0	1870	TCGA
339	I	0	1072	TCGA
340	I	0	669	TCGA
341	I	1	460	TCGA
342	I	0	28	TCGA
343	I	0	674	TCGA
344	II	0	568	TCGA
345	I	1	697	TCGA

346	I	0	800	TCGA
347	I	0	719	TCGA
348	I	0	1239	TCGA
349	I	1	33	TCGA
350	II	0	949	TCGA
351	II	0	1272	TCGA
352	II	0	1071	TCGA
353	I	0	1847	TCGA
354	II	0	739	TCGA
355	I	0	455	TCGA
356	II	0	1216	TCGA
357	I	0	652	TCGA
358	II	1	243	TCGA
359	I	1	719	TCGA
360	I	0	515	TCGA
361	II	0	565	TCGA
362	I	0	541	TCGA
363	I	1	22	TCGA
364	I	0	595	TCGA
365	I	0	79	TCGA
366	I	1	1632	TCGA
367	I	0	476	TCGA
368	I	0	610	TCGA
369	I	1	594	TCGA
370	I	0	2137	TCGA
371	I	0	608	TCGA
372	I	0	1864	TCGA
373	I	0	1305	TCGA
374	I	0	1974	TCGA
375	I	1	210	TCGA
376	II	0	603	TCGA
377	I	0	653	TCGA
378	I	1	2318	TCGA
379	I	0	626	TCGA
380	I	0	2261	TCGA

381	I	0	3635	TCGA
382	I	0	186	TCGA
383	II	1	429	TCGA
384	II	0	839	TCGA
385	II	0	944	TCGA
386	I	0	408	TCGA
387	I	0	48	TCGA
388	I	0	626	TCGA
389	I	0	36	TCGA
390	I	1	1073	TCGA
391	I	1	976	TCGA
392	II	1	187	TCGA
393	I	0	182	TCGA
394	I	1	457	TCGA
395	I	0	658	TCGA
396	I	1	1288	TCGA
397	I	0	1559	TCGA
398	I	1	1167	TCGA
399	I	1	1026	TCGA
400	I	1	826	TCGA
401	I	1	1653	TCGA
402	I	1	1171	TCGA
403	II	0	591	TCGA
404	I	0	1233	TCGA
405	I	0	486	TCGA
406	I	0	610	TCGA
407	I	0	845	TCGA
408	I	0	845	TCGA
409	II	0	1289	TCGA
410	I	0	596	TCGA
411	I	0	218	TCGA
412	I	0	791	TCGA
413	I	1	1798	TCGA
414	I	0	539	TCGA
415	I	1	1229	TCGA

416	I	0	427	TCGA
417	I	0	186	TCGA
418	I	0	1159	TCGA
419	II	0	179	TCGA
420	I	0	741	TCGA
421	II	0	1246	TCGA
422	II	0	791	TCGA
423	I	0	2515	TCGA
424	I	0	505	TCGA
425	I	0	1965	TCGA
426	I	1	300	TCGA
427	I	0	287	TCGA
428	I	0	2077	GEO
429	I	0	1949	GEO
430	I	0	1843	GEO
431	I	0	1834	GEO
432	I	0	1779	GEO
433	I	0	1703	GEO
434	I	0	1693	GEO
435	II	0	1692	GEO
436	I	0	1685	GEO
437	II	0	1652	GEO
438	I	0	1652	GEO
439	I	1	1629	GEO
440	II	0	1623	GEO
441	I	0	1616	GEO
442	I	0	1599	GEO
443	I	0	1596	GEO
444	I	0	1569	GEO
445	II	1	1560	GEO
446	I	0	1554	GEO
447	I	0	1551	GEO
448	I	0	1510	GEO
449	I	0	1507	GEO
450	II	0	1494	GEO

451	I	0	1488	GEO
452	I	0	1488	GEO
453	I	0	1481	GEO
454	I	0	1465	GEO
455	I	0	1402	GEO
456	I	0	1388	GEO
457	I	0	1363	GEO
458	I	1	1357	GEO
459	I	0	1329	GEO
460	I	0	1326	GEO
461	II	0	1293	GEO
462	I	1	1293	GEO
463	II	0	1260	GEO
464	I	0	1240	GEO
465	I	0	1239	GEO
466	I	0	1239	GEO
467	I	0	1238	GEO
468	I	1	1231	GEO
469	I	0	1189	GEO
470	II	0	1174	GEO
471	I	0	1166	GEO
472	II	0	1156	GEO
473	I	0	1155	GEO
474	I	0	1153	GEO
475	I	0	1150	GEO
476	I	0	1147	GEO
477	I	0	1146	GEO
478	I	0	1141	GEO
479	I	1	1135	GEO
480	I	0	1134	GEO
481	I	0	1130	GEO
482	I	0	1127	GEO
483	II	0	1120	GEO
484	I	1	1119	GEO
485	II	0	1112	GEO

486	I	0	1110	GEO
487	I	0	1109	GEO
488	II	0	1106	GEO
489	I	0	1089	GEO
490	I	0	1086	GEO
491	I	0	1085	GEO
492	I	0	1079	GEO
493	I	0	1076	GEO
494	I	0	1074	GEO
495	I	0	1070	GEO
496	II	0	1063	GEO
497	I	0	1061	GEO
498	I	0	1061	GEO
499	II	0	1057	GEO
500	I	0	1057	GEO
501	II	0	1053	GEO
502	I	1	1053	GEO
503	I	0	1042	GEO
504	I	0	1038	GEO
505	I	1	1037	GEO
506	I	0	1035	GEO
507	I	0	1033	GEO
508	I	0	1033	GEO
509	I	1	1031	GEO
510	I	0	1029	GEO
511	I	0	1029	GEO
512	I	0	1025	GEO
513	II	1	1025	GEO
514	I	0	1024	GEO
515	II	0	1019	GEO
516	I	0	1015	GEO
517	I	0	1012	GEO
518	I	0	1011	GEO
519	II	0	1006	GEO
520	I	1	999	GEO

521	II	0	997	GEO
522	I	0	994	GEO
523	II	0	988	GEO
524	I	0	987	GEO
525	I	0	987	GEO
526	I	0	982	GEO
527	I	0	980	GEO
528	II	0	978	GEO
529	II	0	972	GEO
530	I	0	971	GEO
531	I	0	969	GEO
532	I	1	968	GEO
533	I	0	962	GEO
534	I	0	962	GEO
535	I	0	959	GEO
536	I	0	956	GEO
537	I	1	951	GEO
538	I	0	950	GEO
539	II	0	949	GEO
540	I	0	947	GEO
541	I	0	939	GEO
542	I	0	938	GEO
543	I	0	937	GEO
544	I	0	937	GEO
545	I	1	936	GEO
546	I	1	932	GEO
547	I	0	926	GEO
548	I	0	925	GEO
549	I	0	924	GEO
550	I	1	922	GEO
551	I	0	921	GEO
552	I	0	919	GEO
553	I	1	917	GEO
554	I	0	913	GEO
555	I	0	911	GEO

556	I	0	911	GEO
557	II	0	907	GEO
558	I	0	906	GEO
559	I	0	906	GEO
560	I	0	905	GEO
561	I	0	900	GEO
562	I	0	897	GEO
563	II	0	897	GEO
564	I	0	895	GEO
565	I	0	891	GEO
566	I	0	890	GEO
567	I	0	889	GEO
568	I	0	882	GEO
569	I	0	882	GEO
570	I	0	878	GEO
571	I	0	874	GEO
572	II	1	871	GEO
573	I	0	871	GEO
574	I	0	868	GEO
575	I	0	868	GEO
576	I	0	867	GEO
577	I	0	861	GEO
578	I	0	860	GEO
579	I	0	858	GEO
580	I	0	855	GEO
581	II	0	854	GEO
582	I	0	852	GEO
583	I	0	850	GEO
584	I	0	849	GEO
585	I	0	848	GEO
586	I	0	844	GEO
587	I	0	840	GEO
588	II	0	840	GEO
589	I	1	837	GEO
590	I	0	834	GEO

591	I	0	833	GEO
592	I	0	833	GEO
593	I	0	832	GEO
594	I	0	831	GEO
595	I	0	831	GEO
596	I	0	830	GEO
597	I	0	830	GEO
598	II	0	830	GEO
599	I	0	825	GEO
600	I	0	823	GEO
601	I	0	821	GEO
602	I	0	820	GEO
603	I	0	818	GEO
604	I	0	818	GEO
605	I	0	809	GEO
606	I	1	809	GEO
607	I	0	807	GEO
608	I	0	807	GEO
609	I	0	807	GEO
610	II	0	806	GEO
611	II	0	802	GEO
612	II	0	799	GEO
613	I	0	799	GEO
614	I	0	798	GEO
615	I	0	797	GEO
616	I	0	785	GEO
617	I	1	780	GEO
618	I	0	777	GEO
619	I	0	776	GEO
620	I	0	771	GEO
621	I	0	766	GEO
622	I	1	762	GEO
623	I	0	760	GEO
624	I	0	759	GEO
625	II	0	757	GEO

626	I	0	751	GEO
627	I	0	750	GEO
628	I	0	749	GEO
629	I	0	740	GEO
630	I	0	734	GEO
631	I	0	733	GEO
632	II	1	732	GEO
633	I	0	732	GEO
634	I	0	730	GEO
635	II	0	724	GEO
636	I	1	713	GEO
637	I	0	708	GEO
638	I	0	708	GEO
639	I	0	701	GEO
640	I	0	701	GEO
641	I	0	698	GEO
642	II	0	698	GEO
643	I	1	696	GEO
644	I	1	693	GEO
645	I	0	692	GEO
646	I	0	683	GEO
647	I	0	681	GEO
648	I	1	680	GEO
649	II	0	680	GEO
650	I	0	679	GEO
651	II	0	679	GEO
652	I	0	676	GEO
653	II	1	676	GEO
654	I	0	672	GEO
655	II	0	671	GEO
656	I	1	652	GEO
657	I	1	651	GEO
658	I	0	642	GEO
659	I	0	628	GEO
660	I	1	627	GEO

661	I	0	622	GEO
662	I	0	619	GEO
663	II	1	618	GEO
664	I	0	607	GEO
665	II	1	600	GEO
666	I	0	597	GEO
667	I	0	595	GEO
668	II	1	591	GEO
669	I	0	591	GEO
670	I	0	588	GEO
671	I	0	582	GEO
672	I	0	579	GEO
673	I	0	578	GEO
674	I	0	577	GEO
675	II	0	577	GEO
676	I	0	570	GEO
677	I	0	568	GEO
678	I	0	557	GEO
679	I	0	555	GEO
680	I	0	554	GEO
681	I	1	541	GEO
682	I	0	540	GEO
683	II	0	534	GEO
684	I	1	528	GEO
685	II	1	524	GEO
686	I	1	520	GEO
687	II	1	512	GEO
688	I	1	508	GEO
689	I	1	496	GEO
690	I	0	489	GEO
691	II	1	489	GEO
692	I	1	488	GEO
693	II	1	487	GEO
694	I	0	468	GEO
695	I	1	460	GEO

696	II	1	430	GEO
697	I	0	406	GEO
698	I	0	392	GEO
699	I	1	382	GEO
700	I	0	373	GEO
701	I	1	358	GEO
702	I	0	356	GEO
703	I	0	349	GEO
704	I	1	347	GEO
705	I	1	336	GEO
706	I	1	332	GEO
707	I	1	325	GEO
708	II	0	325	GEO
709	I	1	310	GEO
710	I	1	307	GEO
711	I	1	306	GEO
712	II	0	305	GEO
713	I	0	297	GEO
714	I	1	293	GEO
715	II	1	283	GEO
716	II	1	281	GEO
717	I	0	275	GEO
718	II	1	267	GEO
719	II	1	244	GEO
720	II	1	239	GEO
721	II	1	223	GEO
722	I	1	219	GEO
723	I	1	216	GEO
724	I	0	142	GEO
725	I	0	140	GEO
726	I	0	124	GEO
727	I	1	116	GEO
728	II	1	98	GEO
729	I	1	80	GEO
730	II	0	79	GEO

731	I	1	76	GEO
732	II	0	76	GEO
733	II	1	58	GEO
734	II	1	50	GEO
735	II	1	46	GEO
736	I	0	43	GEO
737	I	1	40	GEO
738	II	1	35	GEO
739	I	0	27	GEO
740	I	0	24	GEO
741	I	0	22	GEO
742	I	0	20	GEO
743	I	1	17	GEO
744	I	0	16	GEO
745	I	0	5	GEO
746	I	1	4	GEO
747	II	0	4	GEO
748	II	1	3	GEO

Table S2. Oligonucleotides of siRNAs.

siKRT8-1	GTGGAGAGCTGGCCATTAA
siKRT8-2	TCACCGCAGTTACGGTCAA
siKRT8-3	CTCGAAGCAACATGGACAA
siPERP-1	GTCAGAGCCTCATGGAGTA
siPERP-2	GCTGTCACTTACATCTATA
siPERP-3	CCCAGATGCTTGTCTTCCT

Table S3. The details of the antibodies.

The details of the antibodies used in this study are: Anti-PERP (bs-10783R, bioss, China), Anti-KRT8(bsm-52419R, bioss, China), Anti-GAPDH (D16H11, CST, USA).
